# Ground-Based NDVI Network: Early Validation Practice with Sentinel-2 in South Korea

**DOI:** 10.3390/s24061892

**Published:** 2024-03-15

**Authors:** Junghee Lee, Joongbin Lim, Jeongho Lee, Juhan Park, Myoungsoo Won

**Affiliations:** 1 Forest ICT Research Center, National Institute of Forest Science, Seoul 02455, Republic of Korea; junghee23@korea.kr (J.L.); forestfire@korea.kr (M.W.); 2 Interdisciplinary Program in Agricultural and Forest Meteorology, Seoul National University, Seoul 08826, Republic of Korea; jngh02@snu.ac.kr; 3 National Center for Agro-Meteorology, Seoul 08826, Republic of Korea; jhpark@ncam.kr

**Keywords:** multispectral sensor network, NDVI, validation practice, sentinel-2

## Abstract

As satellite launching increases worldwide, uncertainty quantification for satellite data becomes essential. Misunderstanding satellite data uncertainties can lead to misinterpretations of natural phenomena, emphasizing the importance of validation. In this study, we established a tower-based network equipped with multispectral sensors, SD-500 and SD-600, to validate the satellite-derived NDVI product. Multispectral sensors were installed at eight long-term ecological monitoring sites managed by NIFoS. High correlations were observed between both multispectral sensors and a hyperspectral sensor, with correlations of 0.76 and 0.92, respectively, indicating that the calibration between SD-500 and SD-600 was unnecessary. High correlations, 0.8 to 0.96, between the tower-based NDVI with Sentinel-2 NDVI, were observed at most sites, while lower correlations at Anmyeon-do, Jeju, and Wando highlighting challenges in evergreen forests, likely due to shadows in complex canopy structures. In future research, we aim to analyze the uncertainties of surface reflectance in evergreen forests and develop a biome-specific validation protocol starting from site selection. Especially, the integration of tower, drone, and satellite data is expected to provide insights into the effect of complex forest structures on different spatial scales. This study could offer insights for CAS500-4 and other satellite validations, thereby enhancing our understanding of diverse ecological conditions.

## 1. Introduction

As technology advances, the number of satellites launched worldwide is rapidly increasing every year. Satellites play a crucial role in monitoring various phenomena on Earth. Uncertainty quantification, along with quality assurance and quality control, is crucial for satellite data, as it can be affected by atmospheric effects [1,2], the Sun–target–satellite angle [3,4], and heterogeneous landscapes [5]. These factors can create differences between satellite images and actual phenomena, potentially leading to incorrect interpretations. Validation methods of satellite data can be distinguished into direct and indirect ways. Direct validation involves comparing satellite data with ground observation or field campaign measurements. For example, gross primary productivity derived from the Moderate Resolution Imaging Spectroradiometer (MODIS) can be validated by comparing it with eddy covariance flux tower data [6,7]. Indirect methods cover comparisons with physical models, data-driven products using artificial intelligence or machine learning, and consistency assessments with existing data. However, it is helpful to use multiple data sources for understanding the uncertainties inherent in satellite-derived products, rather than relying on a single source. For instance, in evaluating the accuracy of satellite Normalized Difference Vegetation Index (NDVI) data, it is recommended not only to conduct a direct validation with field observation data but also to employ indirect methods [8], such as the utilization of phenological information [9,10,11]. However, direct validation remains crucial, and the importance of generating and utilizing ground-based data will continue to increase over time.

Vegetation indices have been crucial tools for monitoring the Earth’s vegetation status over the past several decades. Among these indices, NDVI has been a significant indicator, representing plant health [12,13], productivity [14,15,16], and biodiversity [17,18,19,20]. Various satellites, including NASA’s MODIS, the European Space Agency’s Sentinel-3 OLCI (Ocean and Land Colour Instrument), the Landsat series, and NOAA’s AVHRR (Advanced Very High Resolution Radiometer), officially provide NDVI data. These satellite missions constantly monitor changes in surface vegetation and offer long-term data that are crucial for environmental change research. Due to the limited lifespan of satellites, it is essential to ensure consistency among different satellite sensors for long-term vegetation index trend analysis [21,22]. The validation of various satellite-derived NDVI products is necessary to quantify the uncertainty of each product, and sensor calibration may be required if necessary. Comparisons and integration across different satellite sensors, as well as understanding and correcting for the impacts of atmospheric and other environmental factors, necessitate ground observation data from diverse geographical and environmental conditions.

The Committee on Earth Observation Satellites (CEOS) was established in 1984 as an international coordinator for space-based Earth observation activities and aims to facilitate the exchange of Earth observation data. CEOS is organized into two main operational groups: Virtual Constellations and Working Groups (WG). CEOS Virtual Constellations coordinates the global Earth-observing satellite programs among space agencies, supporting key societal information needs. Four prototypal virtual constellations are currently ongoing by CEOS space agencies, namely the Precipitation Constellation, the Land Surface Imaging Constellation, the Atmospheric Composition Constellation, and the Ocean Surface Topography Constellation. CEOS Working Groups are categorized into (1) Calibration and Validation (WGCV), (2) Information Systems and Services (WGISS), and (3) Education, Training, and Capacity Building (WGEDU). In particular, the Land Product Validation (LPV) subgroup of the GEOS WGCV offers guidelines for four validation stages (Table 1) with definitions and current states. Within the CEOS LPV Vegetation Index (VI) focus area, the current MODIS VI product has achieved the highest level of validation (level 3) among the VI outputs. The CEOS LPV VI focus area is developing a good practice protocol for validation considering the uncertainty of the VI, variations in the VI under actual vegetation condition changes, and long-term stability of the VI time series. The group also recommends including the product quality assurance information, uncertainty information through validation, and inter-comparison results.

The Compact Advanced Satellite 500-4 (CAS500-4), scheduled to launch in 2025, will have a revisit period of 3 days, a spatial resolution of 5 m, and will be equipped with multispectral sensors including blue, green, red, red edge, and near-infrared bands. South Korea has a rough terrain with various climate zones distributed within a small area, resulting in the vegetation distribution being heterogeneous. Therefore, using CAS500-4 or Sentinel-2, which have a spatial resolution of about 5 to 10 m, is suitable for monitoring vegetation in South Korea. Additionally, due to the complex topography in Korea, topographic or atmospheric correction may have uncertainties [23,24], so a direct comparison is more appropriate than the indirect comparison of vegetation indices. In this study, we established a ground observation network to validate the satellite-derived NDVI in Korea. The validation sites were designed considering the topographic and vegetative characteristics of Korea, and multi-spectral sensors were installed within the network to reduce costs. Before developing a good practice protocol for NDVI based on CAS500-4 in Korea, early validation practices were conducted using NDVI derived from Sentinel-2, which has similar specifications to CAS500-4. Section 2 introduces the multispectral sensor-based ground observation network we developed, while Section 3 details the methodology for early validation with Sentinel-2. Section 4 and Section 5 present the analysis and discussion of our findings from comparing the ground-observed NDVI with hyperspectral data, analyzing the time series of NDVI, and validating for satellite-derived NDVI. The paper concludes in Section 6. This research helps to understand the uncertainty of satellite-based vegetation indices across different vegetation types. Furthermore, it can also contribute to estimating uncertainties in NDVI products such as Landsat or VIIRS, which have lower resolution than Sentinel-2.

## 2. Ground-Based NDVI Networks

### 2.1. Site Selection

**Figure 1:**
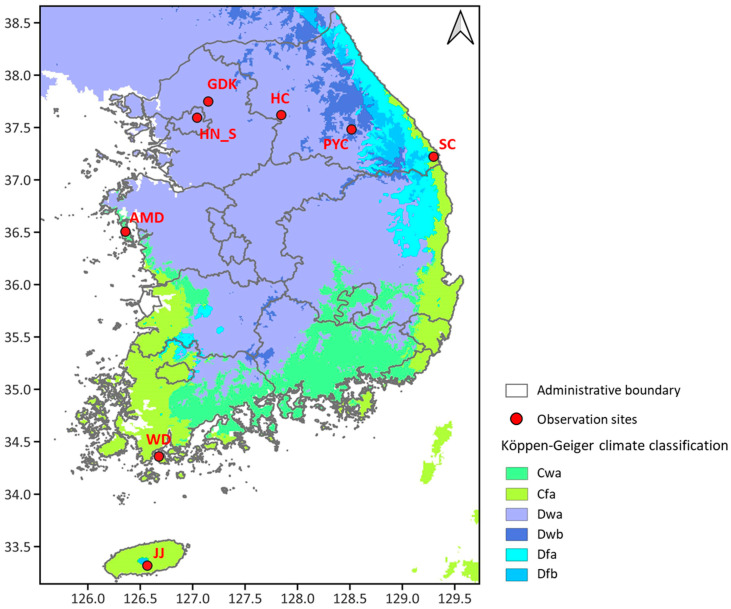
Ground-based observation sites (red points) for calibration/validation in South Korea, with the Köppen–Geiger climate classification [25] in the background. The climatic classifications are abbreviated as follows: Cwa (monsoon-influenced humid subtropical climate), Cfa (humid subtropical climate), Dwa (monsoon-influenced hot-summer humid continental climate), Dwb (monsoon-influenced warm-summer humid continental climate), Dfa (hot-summer humid continental climate), and Dfb (warm-summer humid continental climate).

In this study, the criteria for establishing validation sites considered (1) homogeneity of land cover, (2) easy access and linkage with long-term ecological observation data, and (3) suitability for long-term operational use. We prioritized regions where the National Institute of Forest Science (NIFoS) conducts long-term ecological research. These long-term ecological research sites are advantageous because of their cost-effective, long-term operational capabilities and the ability to use existing ecological data (e.g., plant phenology, leaf area index, forest height, and biomass) for analytical purposes. Eight ground observation sites (Table 2) were selected across the country where eddy covariance towers were installed within the long-term ecological monitoring research sites of NIFoS. Ultimately, more than thirty towers will be installed, taking into account the diverse forest types in South Korea (Figure 1).

### 2.2. Sensor Specifications

Surface reflectance data were achieved using a light-emitting diode (LED)-based multispectral sensors, developed by SolDan Inc. (Seoul, Republic of Korea). The SD-500 sensor was set up at seven sites, except for SC, between 2021 and 2022. The SD-500 sensor is equipped with four spectral bands―blue, green, red, and near-infrared (NIR). It is designed to operate within a temperature range of −35 °C to 50 °C, covering the lowest and highest air temperatures in South Korea. Further specifications are summarized in Table 3. The developed multispectral sensor has been proven to be cost effective for its use in agricultural fields, grasslands, and forests compared to the traditional, more expensive spectrometers [26,27,28,29]. Recently, the SD-500 sensors were replaced with the SD-600 and installed at all eight sites in 2023. The SD-600 sensor, equipped with the AS7343 model (https://ams-osram.com/products/sensors/ambient-light-color-spectral-proximity-sensors/ams-as7343-spectral-sensor (accessed on 10 January 2024)) with the addition of the red edge band, is produced by the ams-OSRAM AG company. The spectral wavelengths of the SD-500 and SD-600, as detailed in Table 4, were configured to match those of Sentinel-2 as well as the CAS500-4, which is scheduled for launch in 2025. Detailed information about the sensor replacement schedule is provided in the last column of Table 2. 

### 2.3. Footprint Coverage for Validation

According to the CEOS Calibration and Validation guidelines (Table 1), it is essential to set the footprint of the sensor installed at the validation site. The sensor footprint should be determined with consideration for the spatial resolution of the satellite being validated, typically set to at least twice the resolution of the satellite image. Given the target spatial resolution of CAS500-4 of 5 m, a minimum radius for a sensor footprint of 10 m is recommended. The footprint of a sensor mounted on a tower is influenced by the tower’s height. Within a 180° field of view, the most intensively sampled area lies within nadir view angles from 0° to ±45°. Under the assumption of an average tree height of 20 m and a tower height of 40 m, the footprint radius of our sensor is calculated to be 20 m. Consequently, the footprint of our ground observation data would be 40 m × 40 m.

## 3. Validation Practice of Sentinel-2 NDVI

### 3.1. Data Processing 

Multispectral sensors, mounted on a tower, measured the surface reflectance at consistent one-minute intervals (Table 2). The tower-based NDVI was calculated using the red and NIR spectral bands of these multispectral sensors. Daily NDVI data were obtained by averaging the per-minute data from 11:00 to 11:20 local time, consistent with the Sentinel-2 overpass time. We acquired hyperspectral sensor data for the JJ and WD sites for the year 2022, coinciding with the observation period of the multispectral sensor. The observations from the hyperspectral sensor were aligned with the red and NIR spectral bands of the multispectral sensor before calculating NDVI. Considering the Sentinel-2 overpassing time, the NDVI values observed at 11 a.m. were used because the observation interval of the hyperspectral sensor was half-hourly. 

To ensure data quality, we implemented three-step temporal filtering processes to exclude outliers: (1) Outliers deviating more than ±2 standard deviations from the average NDVI over the entire period were filtered out. (2) NDVI values exceeding ±2 standard errors from the 28-day moving window-based linear regression were removed. (3) NDVI values exceeding ±1 standard error from the 7-day moving window-based linear regression were eliminated.

The validation exercise was conducted with data acquired in 2022 and 2023 using a Sentinel-2 L2A product, which has specifications similar to those of CAS500-4. The Sentinel-2 NDVI was calculated as NDVI = (Band8 − Band4)/(Band 8 + Band 4) using its red and NIR reflectance bands. Sentinel-2 images from 2022 to 2023 were processed on the Google Earth Engine platform, filtering out low-quality pixels—such as clouds, cloud shadows, and topographic shadows—based on the scene classification layer. The Sentinel-2 NDVI with different buffer radii―5 m, 10 m, 15 m, 20 m, 25 m, and 30 m―were extracted from each site in Table 2 to analyze whether the variance in Sentinel-based NDVI within the tower footprint influences validation.

### 3.2. Evaluation Metrics

The Sentinel-2 NDVI, within buffers of 5 m, 10 m, 15 m, 20 m, 25 m, and 30 m from the tower center, were quantitatively assessed with tower observation data. After matching the ground observations with Sentinel-2 NDVI by date, statistical measures such as mean absolute error (MAE; Equation (1)), mean bias error (MBE; Equation (2)), root mean squared error (RMSE; Equation (3)), and relative root mean squared error (RRMSE; Equation (4)) were used for evaluation.

(1) $$MAE=\frac{1}{n}\sum_{i=1}^{n}\left|y_{i}-\hat{y_{i}}\right|$$

(2) $$MBE=\frac{1}{n}\sum_{i=1}^{n}\left(y_{i}-\hat{y_{i}}\right)$$

(3) $$RMSE=\sqrt{\frac{1}{n}\sum_{i=1}^{n}{\left(y_{i}-\hat{y_{i}}\right)}^{2}}$$

(4) $$RRMSE=\sqrt{\frac{\frac{1}{n}\sum_{i=1}^{n}{\left(y_{i}-\hat{y_{i}}\right)}^{2}}{\sum_{i=1}^{n}{\left(\hat{y_{i}}\right)}^{2}}}$$

## 4. Results

### 4.1. Comparison between SD-500 and SD-600 Sensors

**Figure 2:**
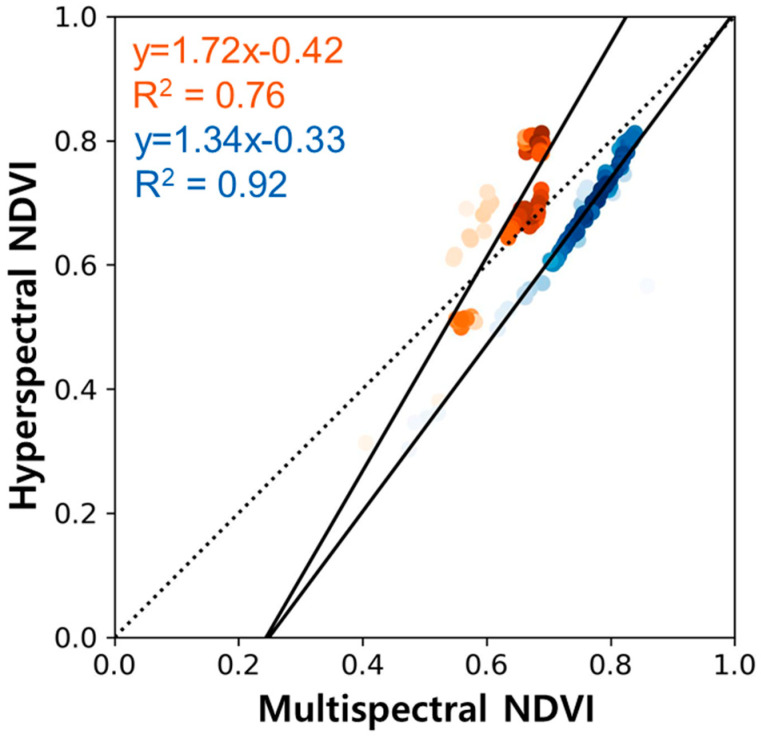
Comparison of multispectral sensor-based NDVI with the hyperspectral sensor-based NDVI. The color intensity deepens with increasing density. Shades of orange represent the SD-500, while shades of blue denote the SD-600.

**Figure 3:**
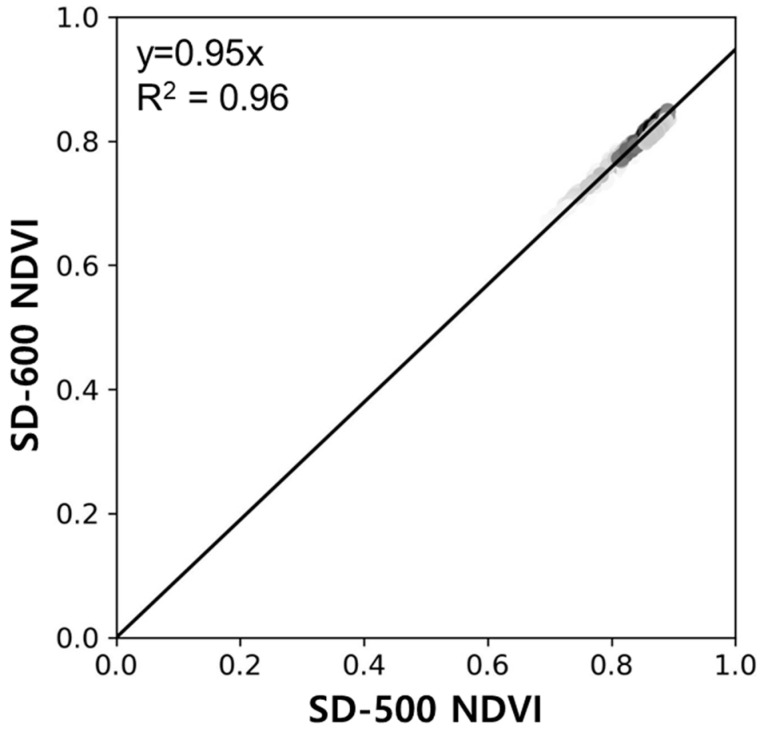
NDVI comparison between SD-500 and SD-600. High point density is represented by black, while the opposite is indicated by light gray.

The daily NDVI derived from two multispectral sensors was assessed against the hyperspectral sensor-based NDVI (Figure 2). Both SD-500- and SD-600-based NDVI values demonstrated a high correlation with the hyperspectral-based NDVI, at 0.76 and 0.92, respectively. Unlike the generally overestimated NDVI from the SD-600, the NDVI derived from the SD-500 tended to be overestimated at values lower than 0.6 and underestimated at values higher than 0.6. For consistency between multispectral sensors, an inter-comparison between SD-500 and SD-600 was conducted (Figure 3). We analyzed the AMD, HC, and WD sites where both SD-500 and SD-600 measurements are available. The data used covered the period from 15 April to 4 December 2023 for AMD, from 26 April to 17 November 2023 for HC, and from 15 April to 17 November 2023 for WD. The slope between the NDVI values obtained from SD-500 and SD-600 sensors was close to 1. At the AMD site, the NDVI values derived from the SD-500 tend to be slightly higher than those from the SD-600 (slope 0.74, R² 0.82, *n* = 78), compared to the HC (slope 0.93, R² 0.99, *n* = 88) and WD (slope 0.82, R² 0.88, *n* = 51). The discrepancy in NDVI at the AMD site appears to stem from the difference in the reflectance of the red band. Specifically, the divergence in surface reflectance within the red band exhibited a stronger correlation of 0.81 with the NDVI differences between SD-500 and SD-600, compared to a correlation coefficient of 0.06 with the NIR band. The lower correlation of the red band between SD-500 and SD-600 may be attributed to the wavelength difference of about 10 nm in the red band. Based on these results, we concluded that both SD-500 and SD-600 sensors could be used interchangeably.

### 4.2. Timeseries of Tower-Based NDVI

**Figure 4:**
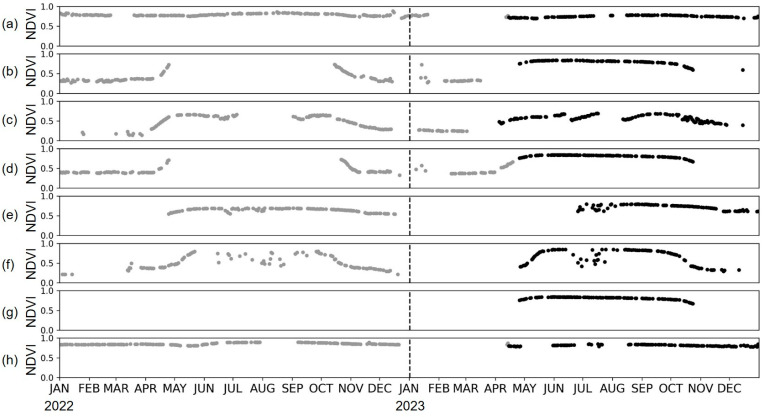
NDVI time series from eight ground observation sites for 2022–2023: (**a**) AMD, (**b**) GDK, (**c**) HN_S, (**d**) HC, (**e**) JJ, (**f**) PYC, (**g**) SC, and (**h**) WD. NDVI values from the SD-500 are presented by gray points, and those from the SD-600 by black points. Outliers beyond one standard deviation from the mean were omitted.

Figure 4 presents the time series of NDVI obtained from the tower-based multispectral sensors across eight sites. The evergreen needle-leaf forest (e.g., AMD) and an evergreen broad-leaf forest (e.g., WD) have stable NDVI values around 0.8 throughout the year, indicating little seasonal fluctuation in these biomes. In contrast, the deciduous broad-leaf forests (e.g., GDK, HN_S, and PYC) and mixed forests (e.g., HC and SC) showed clear phenological cycles, with the exception of JJ, where the proportion of evergreen trees is relatively higher compared to other species. During the growing season, both deciduous broad-leaf forests and mixed forests exhibited NDVI values around 0.8. However, in the leaf off season, the NDVI value of deciduous forests significantly decreases, falling to approximately 0.3, which is lower than that observed in mixed forests.

### 4.3. Validation Experiment with Sentinel-2 NDVI

**Figure 5:**
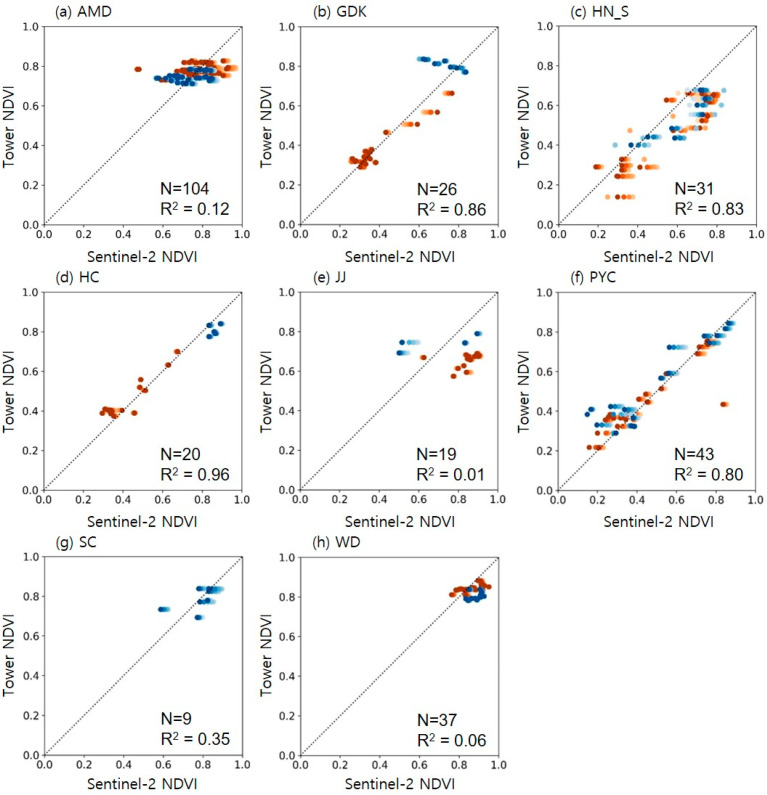
Comparison between Sentinel-2 NDVI and tower-based NDVI on eight sites: (**a**) AMD, (**b**) GDK, (**c**) HN_S, (**d**) HC, (**e**) JJ, (**f**) PYC, (**g**) SC, and (**h**) WD. Shades of orange represent the SD-500, while shades of blue denote the SD-600. The marker color deepens as the buffer radius increases from 5 m to 30 m. The dotted line indicates the 1:1 line.

Sentinel-2 NDVI values, extracted at 5 m intervals from a 5 m to 30 m radius around each tower equipped with multispectral sensors, were compared with tower-based NDVI (Figure 5). There were little differences in NDVI based on the radius size from the center, so subsequent analyses have been described based on the results within a 5 m radius. The correlation between Sentinel-2 NDVI and tower-based NDVI varied by site. The sites with DBF and MF—GDK, HN_S, HC, PYC, and SCs—showed a strong correlation with Sentinel-2 and tower-based observations, achieving R² values between 0.80 and 0.96. In contrast, the sites containing evergreens—AMD, JJ, and WD—exhibited significantly lower correlations, with R^2^ values of 0.12, 0.01, and 0.06, respectively. The low correlations at such sites seem to result from the NDVI values from Sentinel-2 and tower measurements being concentrated on small ranges between 0.6 and 0.8. Overall, bias was negligible, except for HN_S and JJ, which showed a positive bias for Sentinel-2 NDVI as shown in Table 5. Specifically, the bias and errors at the HN_S site increased and decreased relative to the distances from the tower, demonstrating spatial heterogeneity within a 30 m radius. This suggests that it might be better to exclude this site in future validation for satellite-based NDVI. At the JJ site, a significant portion of the data used in the analysis came from the SD-500 (Figure 5e), potentially causing an underestimation of NDVI values at higher ranges. This underestimation likely contributed to the positive bias in Sentinel-2 NDVI measurements at the JJ site, indicating the need for further validation, particularly using SD-600 data. Moreover, at seven sites excluding JJ, the RRMSE was below 5%, indicating excellent validation results. Even at the JJ site, the RRMSE was approximately 5.3%, which still denotes a good result.

## 5. Discussions

To achieve the CEOS Calibration and Validation Stage 3, it is necessary to quantify uncertainties across diverse forest types, topographies, and climates. The tower-based network equipped with multispectral sensors in South Korea can significantly contribute to this uncertainty quantification. The rugged terrain in South Korea is characterized by distinct climate zones and complex forest structures, resulting in spatial heterogeneity. Accordingly, we have been installing multispectral sensors in a bi-hemispherical configuration, suitable for heterogeneous canopies [30]. The statistical significance of our findings is expected to improve with the completion of the installation of more than thirty towers, which will reflect the varied forest conditions across South Korea. For the validation of satellite-derived products, developing a protocol for the multispectral sensors in our network is essential. This protocol must include periodic sensor calibration and meticulous quality control of observation data. Moreover, further comparisons with radiative transfer models and indirect indicators, such as plant phenology, gross primary productivity, and leaf area index, are also necessary.

Matching the footprint between satellite-derived products and ground observations is a crucial factor. This study estimated the sensor footprint size based on the average tree height around the towers and the sensor installation height. The actual footprint is influenced by the radiative transfer path length, which varies with forest structure and atmospheric conditions. Theoretically, 80% of the total radiation entering a hemispherical sensor over flat bare ground falls within a field of view (FOV) of approximately 63.4 degrees, with the maximum contribution within 45 degrees [31]. Ref. [32] reported that chlorophyll fluorescence footprint modeling at towers installed in a bi-hemispherical configuration showed that 90% of total radiation came from an FOV width within 72 degrees. Therefore, in a bi-hemispherical setup, 80–90% of the radiation is expected to come within a FOV of 60–70 degrees. In other words, if the sensor is 15 m above the canopy top, the radius of the footprint would be approximately 25.98 to 41.21 m. When validating Sentinel-2 NDVI within a radius of 5 m to 30 m in 5 m intervals from each tower, no significant differences were observed. In the future, further validation is needed for the surface reflectance of multispectral sensors using drone-based hyperspectral imaging considering the footprint.

**Figure 6:**
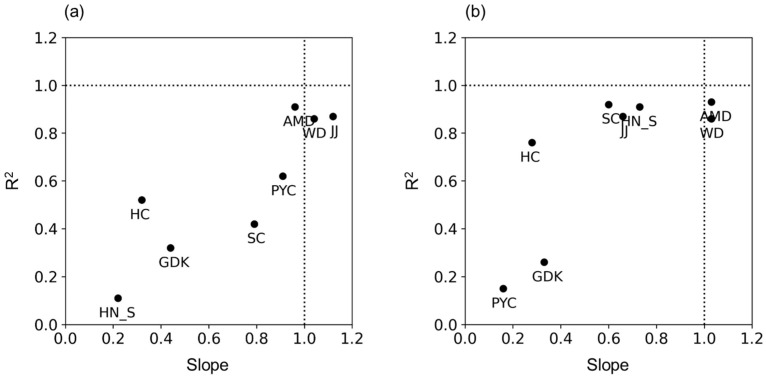
Relationship between the tower-based surface reflectance and its difference with Sentinel-2 for (**a**) red band and (**b**) NIR band. The difference was calculated by differencing between Sentinel-2 and tower-based data. The X-axis indicates slope, and the Y-axis indicates R^2^.

At sites where Sentinel-2 NDVI performance was low, including AMD, JJ, and WD, surface reflectance in the red and NIR bands was observed to be higher compared to other sites, as shown in Figure 6. This discrepancy could be attributed to local environmental factors and the complexity of forest structures, which impact surface reflectance. The influence of shadows, caused by complex structures, introduces uncertainty, highlighting the importance of biome-specific calibration, as [33] has suggested. Furthermore, atmospheric effects, such as Rayleigh and aerosol scattering [34], along with the scattering ratio of the red band being approximately three times higher than that of the NIR band [8], may also contribute to errors. Additional potential causes may include subtle differences in spectra between Sentinel-2 and tower-based multispectral sensors, terrain effects [35,36], canopy shadowing [37], and effects due to canopy clumps [38]. Consequently, future analysis that integrate tower, drone, and satellite data must account for the shadowing effect, particularly emphasizing the need for biome-specific calibration in evergreen forests (e.g., AMD, JJ, and WD).

## 6. Conclusions

In this research, we established a tower-based network equipped with multispectral sensors to validate NDVI products of Sentinel-2. As an early validation practice, we validated the satellite-derived NDVI with eight sites, AMD, GDK, HN_S, HC, JJ, PYC, SC, and WD, which are located on the long-term ecological monitoring research sites of NIFoS. Before validating the satellite-derived product, we compared NDVI between multispectral and hyperspectral data on the Sentinel-2 local overpassing time. The two multispectral sensors, SD-500 and SD-600, showed high correlations of 0.76 and 0.92 with the hyperspectral sensor, respectively. And as the correlation between SD-500 and SD-600 was high (R^2^ of 0.96), calibration between SD-500 and SD-600 was not required for the validation of satellite-derived NDVI. The tower-based NDVI showed high correlations between 0.8 and 0.96 with Sentinel-2 NDVI at most sites, while low correlations were presented for AMD, JJ, and WD sites where evergreen forests were present. Statistically, most sites showed excellent validation results. It is believed that the low performance in evergreen forests is due to complex forest structures, including shadows and canopy clumping, necessitating further research focused on evergreen forests. In this research, we carried out validation practices at eight sites, but the statistical robustness is expected to improve upon the completion of installations at 30 sites. However, there is a need to develop a validation protocol that considers different biomes and canopy structures, beginning with site selection. In addition, the footprint coverage of the tower-based multispectral sensors should be analyzed considering the canopy structure by integrating tower, drone, and satellite data for meticulous analysis. This research is applicable not only to CAS500-4 but also to NDVI validation for other satellites. Given the limited number of current ground observation sites, the network developed in this study offers the potential to facilitate validations across a broader spectrum of ecological conditions.

## Figures and Tables

**Table 1:** Validation stages from CEOS.

	Validation Stage
0	- No validation. Product accuracy has not been assessed. Product considered beta.
1	- Product accuracy is assessed from a small (typically <30) set of locations and time periods by comparison with in situ or other suitable reference data.
2	- Product accuracy is estimated over a significant (typically >30) set of locations and time periods by comparison with reference in situ or other suitable reference data. - Spatial and temporal consistency of the product, and its consistency with similar products, has been evaluated over globally representative locations and time periods. - Results are published in the peer-reviewed literature.
3	- Uncertainties in the product and its associated structure are well quantified over a significant (typically >30) set of locations and time periods representing global conditions by comparison with reference in situ or other suitable reference data. - Validation procedures follow community-agreed-upon good practices. - Spatial and temporal consistency of the product, and its consistency with similar products, has been evaluated over globally representative locations and time periods. - Results are published in the peer-reviewed literature.
4	- Validation results for Stage 3 are systematically updated when new product versions are released or as the interannual time series expands. - When appropriate for the product, uncertainties in the product are quantified using fiducial reference measurements over a global network of sites and time periods (if available).

**Table 2:** Location for ground observation tower. The last column indicates the date when the sensor changed.

Site Name	Site ID	Forest Type *	Latitude	Longitude	Start of Observation	SD500→SD600
Anmyeon-do	AMD	ENF	36.50564506	126.3590538	1 January 2022	15 April 2023
Gwangneung Deciduous forest	GDK	DBF	37.7486814	127.1488721	28 September 2021	17 March 2023
Hongneung	HN_S	DBF	37.59386247	127.0428176	12 March 2021	17 March 2023
Hongcheon	HC	MF	37.61935603	127.8456996	31 March 2021	26 April 2023
Jeju	JJ	MF	33.31797231	126.5677235	31 March 2022	25 June 2023
Pyeongchang	PYC	DBF	37.48184042	128.516041	1 January 2021	25 April 2023
Samcheok	SC	MF	37.22140273	129.2986015	26 April 2023	-
Wando	WD	EBF	34.35953729	126.6777971	26 March 2021	15 April 2023

* DBF: Deciduous Broad-leaf Forest, EBF: Evergreen Broad-leaf Forest, ENF: Evergreen Needle-leaf Forest, MF: Mixed Forest.

**Table 3:** Specification of SD-500 and SD-600 sensors.

Detector Element	Light Emitting Diode, Photodiode	Multi-Spectral Sensor (AS7343)
Spectral Bands	Blue, Green, Red, NIR	Blue, Green, Red, Red Edge, NIR
Operating Temperature Range	−35~50 °C	−30~85 °C
Operating Humidity Range	0~100%	0~100%
Cosine Response	±5% (Zenith Angle within 70°)	±5% (Zenith Angle within 70°)
Diameter	62 mm	62 mm
Height	55 mm	55 mm
Weight	330 g	330 g
Input Voltage	12 VDC	12 VDC

**Table 4:** Central wavelength and band width of SD-500, SD-600, CAS500-4, and Sentinel-2A spectral bands.

Band	SD-500		SD-600		CAS500-4		Sentinel-2A	
	Central Wavelength (nm)	Bandwidth (nm)	Central Wavelength (nm)	Bandwidth (nm)	Central Wavelength (nm)	Bandwidth (nm)	Central Wavelength (nm)	Bandwidth (nm)
Blue	468	59	475	30	490	66	492.4	66
Green	565	42	550	35	560	26	559.8	36
Red	660	44	640	50	665	31	664.6	31
RedEdge	-	-	690	55	705	15	705	15
NIR	850	100	855	54	842	106	832.8	106

**Table 5:** Error metrics of Sentinel-2 NDVI on eight sites. Four metrics are mean bias error (MBE), mean absolute error (MAE), root mean squared error (RMSE), and relative root mean squared error (RRMSE). The rows represent the buffer radius at each tower.

**(a) AMD**					**(b) GDK**				
**Radius**	**MBE**	**MAE**	**RMSE**	**RRMSE(%)**	**Radius**	**MBE**	**MAE**	**RMSE**	**RRMSE(%)**
5 m	−0.008	0.059	0.080	1.022	5 m	−0.018	0.058	0.081	3.103
10 m	−0.005	0.059	0.081	1.030	10 m	−0.021	0.054	0.076	2.902
15 m	0.001	0.060	0.082	1.036	15 m	−0.023	0.054	0.076	2.917
20 m	0.007	0.062	0.084	1.047	20 m	−0.025	0.053	0.076	2.936
25 m	0.011	0.063	0.085	1.054	25 m	−0.028	0.053	0.077	2.968
30 m	0.015	0.064	0.086	1.060	30 m	−0.027	0.054	0.077	2.969
**(c) HN_S**					**(d) HC**				
**Radius**	**MBE**	**MAE**	**RMSE**	**RRMSE(%)**	**Radius**	**MBE**	**MAE**	**RMSE**	**RRMSE(%)**
5 m	0.084	0.100	0.116	3.412	5 m	−0.017	0.049	0.057	2.244
10 m	0.104	0.114	0.129	3.704	10 m	−0.016	0.050	0.057	2.244
15 m	0.114	0.122	0.137	3.869	15 m	−0.015	0.050	0.057	2.242
20 m	0.111	0.126	0.143	4.043	20 m	−0.013	0.049	0.056	2.187
25 m	0.105	0.115	0.130	3.758	25 m	−0.013	0.048	0.055	2.146
30 m	0.079	0.095	0.112	3.391	30 m	−0.013	0.049	0.055	2.157
**(e) JJ**					**(f) PYC**				
**Radius**	**MBE**	**MAE**	**RMSE**	**RRMSE(%)**	**Radius**	**MBE**	**MAE**	**RMSE**	**RRMSE(%)**
5 m	0.135	0.184	0.191	5.367	5 m	−0.034	0.065	0.101	3.137
10 m	0.134	0.183	0.190	5.333	10 m	−0.020	0.056	0.090	2.752
15 m	0.139	0.183	0.190	5.311	15 m	−0.011	0.052	0.087	2.594
20 m	0.143	0.184	0.191	5.319	20 m	−0.005	0.051	0.086	2.532
25 m	0.146	0.184	0.191	5.308	25 m	−0.001	0.051	0.086	2.516
30 m	0.147	0.184	0.191	5.303	30 m	0.001	0.052	0.086	2.524
**(g) SC**					**(h) WD**				
**Radius**	**MBE**	**MAE**	**RMSE**	**RRMSE(%)**	**Radius**	**MBE**	**MAE**	**RMSE**	**RRMSE(%)**
5 m	−0.009	0.040	0.061	2.578	***5 m***	0.039	0.050	0.060	1.133
10 m	0.005	0.046	0.060	2.483	***10 m***	0.042	0.051	0.061	1.139
15 m	0.017	0.053	0.061	2.476	***15 m***	0.043	0.052	0.061	1.144
20 m	0.027	0.059	0.064	2.592	***20 m***	0.044	0.052	0.061	1.148
25 m	0.033	0.063	0.068	2.736	***25 m***	0.044	0.052	0.061	1.150
30 m	0.036	0.066	0.071	2.848	***30 m***	0.043	0.052	0.061	1.149

## Data Availability

The data presented in this study are available on request from the corresponding author.
